# Impact of Be EPIC on Person-Centered Communication

**DOI:** 10.1093/geroni/igaa057.1912

**Published:** 2020-12-16

**Authors:** Marie Savundranayagam, Kristine Williams, Shalane Basque, Joseph Orange, Marita Kloseck, Karen Johnson, Vicki Schmall

**Affiliations:** 1 Western University, London, Ontario, Canada; 2 University of Kansas, Lawrence, Kansas, United States; 3 McCormick Dementia Services, London, Ontario, Canada; 4 Aging Concerns, West Linn, Oregon, United States

## Abstract

This study assessed the impact of Be EPIC, a dementia-focused, person-centered communication intervention for personal support workers (PSWs). Video-recorded conversations between PSWs and simulated persons with dementia assessed whether Be EPIC participants (n=13) (versus a wait-list control group, n=8) reported a greater proportion of person-centered communication utterances (recognition, negotiation, facilitation, validation). We used linear mixed model analysis to investigate if Be EPIC influenced PSWs’ person-centered communication. Group (Be EPIC versus control group), time (pre-, post-, and 3-months) and their interaction were included in the model. There was a significant group by time interaction. Follow-up tests showed that participants who took Be EPIC showed significant increases in person-centered utterances from pre- to post-training and pre-training to 3 months later. Participants in the control group showed no changes in person-centered communication. These findings show that Be EPIC enhanced person-centered communication, which is essential for quality of care.

